# Altered expression of miRNAs and mRNAs reveals the potential regulatory role of miRNAs in the developmental process of early weaned goats

**DOI:** 10.1371/journal.pone.0220907

**Published:** 2019-08-08

**Authors:** Rongrong Liao, Yuhua Lv, Lihui Zhu, Yuexia Lin

**Affiliations:** Institute of Animal Husbandry and Veterinary Science, Shanghai Academy of Agricultural Sciences, Shanghai, China; Institute of Subtropical Agriculture, Chinese Academy of Sciences, CHINA

## Abstract

MicroRNAs (miRNAs) play pivotal roles in growth, development, and stress responses. However, the regulatory function of miRNAs in early weaned goats remains unclear. Deep sequencing comparison of mRNA and miRNA expression profiles showed that 18 miRNAs and 373 genes were differentially expressed in pre- and post-weaning Chongming white goats. Bioinformatics analysis indicated that these differentially expressed genes are involved in cellular processes, developmental processes, and growth in terms of biological process analysis. KEGG analysis showed that downregulated genes were enriched in salivary secretion, bile secretion, vascular smooth muscle contraction, and calcium signaling pathways. Additionally, a miRNA-mRNA co-expression network of the 18 dysregulated miRNAs and their 107 target mRNAs was constructed using a combination of Pearson’s correlation analysis and prediction by miRanda software. Among the downregulated miRNAs, two (chi-miR-206 and chi-miR-133a/b) were muscle development-related and the others were cell proliferation associated. Further RT-qPCR analysis confirmed that downregulated miRNAs (chi-miR-99b-3p, chi-miR-224, and chi-miR-10b-5p) were highly expressed in muscle tissues (heart, spleen, or kidney) of the rapid growth period (7-month old) in Chongming white goats. The results of the present study suggested that weaning induced cell proliferation repression in post-weaning goats, providing new insight into the mechanism of muscle development of goats, although additional details remain to be elucidated in the future.

## Introduction

In China, under the background of continuous optimization of meat production for consumers, the requirements for the quality of goat meat are increasing, especially regarding the demand for goat meat with high protein and low cholesterol. Goat meat is increasingly favored by consumers because of its lower cholesterol content compared with that of sheep meat. Chongming white goats, which belong to the Yangtze River Delta white goat family, are an excellent breed regarding fur and meat, as determined by the unique geographical environment of Chongming island in Shanghai (China). The breed is deeply loved by consumers because of its delicious meat and light taste. Moreover, these goats also have excellent characteristics regarding high fertility and adaptability, making them an ideal source of meat.

In meat goat production, early weaning of lambs is a key technology in intensive feeding mode and is beneficial to the fertility of ewes and the reduction of rearing cost. However, weaning is a dramatic metabolic event for the lambs, as the maturation of the digestive system and immune system of the lambs are incomplete [1]. The isolation from the ewes, changes in diet, and environmental factors inevitably cause psychological and physiological stress on the lambs, resulting in reduced feed intake, repressed indigestion, and even growth retardation after weaning [2,3].

MicroRNAs (miRNAs) are a class of endogenous non-coding single-stranded RNAs involved in the regulation of post-transcriptional genes, and they are the most important members of the family of noncoding RNAs [4]. The growth and development of animal muscle tissue is an extremely complex biological process regulated by a variety of transcription factors, cell signaling molecules, and metabolic pathways, which are also confirmed to be regulated by miRNAs [5,6]. The involvement of miRNAs in sheep muscle growth and development was recently reported [7,8]. Indeed, miRNAs also play pivotal roles in stress responses, including heat stress [9], cold stress [10], as well as weaning stress [11], as studied in other animals. miRNAs are hypersensitive to weaning stress in piglets and calf. In a study, 16, 98, and 22 miRNAs were shown to be altered in the small intestine of piglets at 1, 4, and 7 days after weaning, respectively, and these altered miRNAs are involved in the regulation of metabolism, stressful response, and immune function [11]. In 2016, the miRNA expression profiles in weaned piglets at 4 days post-weaning were compared between the serum and the small intestine by Tao et al. [12]. miRNAs involved in immune and stress response regulation were dysregulated in the serum of weaned piglets. Among the altered miRNAs, miR-194b-5p was downregulated both in the serum and small intestine of piglets induced by weaning stress [12]. The authors also demonstrated that overexpression of miR-146b promoted IPEC-J2 cell apoptosis by targeting toll like receptor 4 [13]. More recently, the miRNA expression profiles in calf rumen were compared between the pre- and post-weaning periods [14], and 122 differentially expressed miRNAs were identified. Further functional analysis revealed that these miRNA were involved in rumen development, immune regulation, and digestion processes, which were significantly regulated by bta-miR-145 and bta-miR-199a-3p. The authors also compared the miRNA expression profiles in the calf ileum between the two periods, and identified several miRNA-mRNA pairs such as bta-miR-374a-FBXO18, bta-miR-374a-GTPBP3, and bta-miR-374a-GNB2, which played crucial roles in ileum development, and bta-miR-15b-IKBKB, which was involved in immune functions [15]. These studies provided important information suggesting that the altered miRNAs caused by weaning stress were associated with tissue development and the development of the immune system. However, compared with other animals, research on goat miRNAs is lagging, and few reports on weaning stress and goat development are available.

Furthermore, it is currently believed that miRNAs in circulating blood are tissue or cellular origin, which can be transported to recipient cells and regulate the association between physiological activity and disease through their circulating form [16,17]. In the present study, we integrated mRNA and miRNA profiles to identify weaning stress-responsive miRNAs, providing important information regarding the involvement of miRNAs in muscle development in goats.

## Methods and materials

### Ethics statement

This study was carried out in accordance with the recommendations of Guidelines for Experimental Animals established by the Shanghai Academy of Agricultural Sciences (Shanghai, China). The protocol was approved by the Shanghai Academy of Agricultural Sciences Animal Ethics Committee.

### Experimental animals and sample collection

The goats used in this experiment were raised in Chongming white goat farm of Shanghai academy of agricultural sciences, Chongming district, Shanghai. Twelve single reared male lambs of the Chongming white goat breed with an average body weight of 9.53 ± 0.39 kg were chose for the study. The lambs were separated from the ewes and weaned at 45 days of age. Each lamb were collected 3 mL venous jugular blood at 1 day before weaning (at 44 day of age) and 3 days after weaning (48 day of age), respectively. All samples were immediately snap-frozen in liquid nitrogen and stored at -80 °C for further RNA extraction.

### Analysis of serum biological indices between weaned and control goats

Serum samples were isolated by centrifugation at 3,500 g for 15 min at 4°C (5415R, Eppendorf, Germany). The antioxidant indices, including superoxide dismutase (SOD) and reduced glutathione (GSH) were analyzed according to the protocol provided by the manufacturer (Nanjing Jiancheng Bioengineering Institute, Nanjing, China). Triglyceride (TG), albumin (ALB), and cholesterol (T-CHO) concentrations were determined on the automatic biochemical analyzer (AU5800, Beckman, MN, USA).

### RNA isolation and sequencing

Total RNA from whole blood (0.5  mL) was extracted using the TRIzol reagent (Life Technologies, Carlsbad, CA, USA) as suggested by the manufacturer. The extracted RNA was quantified on Nanodrop 2000 spectrophotometer (Thermo Scientific, Willmington, DE, USA), and equal amounts of RNA from four goats were further pooled for small RNA and mRNA library generation. Each library contained miRNAs or mRNAs pooled from four goats, therefore three pre- and three post-weaning small RNA libraries were constructed, three pre- and three post-weaning mRNA libraries were constructed similarly. Next generation sequencing was performed on an Illumina Hiseq 4000 machine at Shanghai Majorbio Bio-pharm Technology Co., Ltd (Shanghai, China).

Small RNA libraries (six) were created by using the TruSeq Small RNA Library Prep kit (Illumina, San Diego, CA, USA) according to the manufacturer’s instruction. In brief, 1 μg pooled total RNA was first ligated with 3′ and 5′ adapters. Reverse transcription was then performed to generate cDNA using random primers provided by the manufacturer. The amplified cDNA was purified on 6% Novex TBE PAGE gels and evaluated by Picogreen assay (Life Technologies, Carlsbad, CA, USA). Sequencing reads and miRNA identification were performed as described previously [18]. Conserved known miRNAs were identified through mapping to the precursor miRNAs obtained from miRBase v.21. The miRDeep2 algorithm was used to identify novel miRNA candidates. Differentially expressed miRNAs were computed using the unpaired *t*-test with a cut off |log2FC| > = 1 and *P*-value <0.05.

For mRNA sequencing, the Truseq RNA sample prep Kit (Illumina, San Diego, CA, USA) was used for mRNA library creation according to the instructions. Briefly, mRNAs purified from total RNA were randomly broken into 300 bp small fragments. First and second strand cDNA synthesis was performed using random hexamers (Illumina, San Diego, CA, USA) according to the manufacturer’s instructions. The bead-bound cDNA was digested by endonuclease NlaIII, and the 3’cDNA bound fragments were precipitated and added with the Illumina adaptor 1 and 2, and the products were prepared for sequencing. Normalized gene expression levels were calculated by using RSEM (http://deweylab.github.io/RSEM/). Then, the DESeq2 program was used for the differentially expressed genes (DEGs) between the two groups, with the threshold of significance set at *P* < 0.05 & |log2FC| > = 1. GO and pathway analysis of the DEGs were perform based on GO (Gene Ontology, http://www.geneontology.org/) and KEGG (Kyoto encyclopedia of genes and genomes pathways, http://www.genome.jp/kegg/).

### Integrative analysis of miRNA and mRNA data

The goat genome (Capra hircus ARS1) was used to identify the known and novel miRNAs. First, miRNA target prediction was performed by using the prediction algorithm miRanda. The predicted targets and the DEGs were compared to obtain genuine miRNA targets, and the overlapped genes were identified. Finally, Pearson’s correlation analysis was performed to identify negatively correlated miRNA-mRNA pairs using the sequencing data from the same lambs. Significant interactions were identified by negative correlation coefficients >0.7 and *P* values of < 0.05. The interaction network between the differentially expressed miRNA and targets related to the DEGs were visualized by Cytoscape [19]. The datasets for this study can be found in the NCBI SRA database with Bioproject accession numbers PRJNA507343 and PRJNA507087.

### Validation of miRNA and mRNA profiles by qRT-PCR

For mRNA and mature miRNA quantification, first-strand cDNA was synthesized using HiScript II Q RT SuperMix for qPCR (Qiagen, Germany) and the miScript II Reverse Transcription Kit (Qiagen, Germany), respectively, as suggested by the manufacturer. A QuantiFast SYBR Green PCR Kit (Qiagen, Germany) was used for qRT-PCR analysis with the following conditions: 5 min at 95°C followed by 40 cycles of 10s at 95°C, and 30s at 60°C. Goat GAPDH and U6 RNA were used as internal standards for mRNA and miRNA detection, respectively. All reactions were run in triplicate and calculated by the 2^−ΔCt^ method [20]. Primers were shown in **S1 Table**.

### Expression analysis of selected miRNAs in different tissues of goats

Generally, Chongming white goats grow fast from 4 to 8 months and slowly after 18 months of age. Hence, at 7 and 24 months of age respectively, three healthy male goats were selected and humanely euthanized with CO_2_ inhalation for tissue sample collection. Other goats were humanely raised together. Total RNA from the heart, liver, spleen, kidney, and skeletal muscle (pectoral muscles) tissues was extracted by using TRIzol Reagent (Life Technologies, Carlsbad, CA, USA) according to the total RNA extraction protocol. The extracted RNA was quantified on Nanodrop 2000 spectrophotometer (Thermo Scientific, Willmington, DE, USA). The expression data of selected miRNAs were obtained by the same method described above for miRNA qRT-PCR analysis. Differences were considered statistically significant at *P* < 0.05.

## Results

### Weaning induced dysregulation of miRNAs and mRNAs

To evaluate the effect of weaning stress on miRNA and mRNA expression in Chongming white goats, miRNA and mRNA profiles were investigated in pre- (Control) and post-weaning (Weaned) goats by sequencing. Pre-weaning goats were used as controls. The workflow of these analyses is summarized in **S1 Fig**. Eighteen significantly differentially expressed miRNAs (8 upregulated, 10 downregulated) were identified in the goats after weaning (**Table 1, S2 Fig**). Chi-miR-206, chi-miR-99b-3p, chi-miR-224, chi-miR-143, and chi-miR-133a/b were the top five significantly downregulated miRNAs. Chi-miR-206 and chi-miR-133a/b are involved in the regulation of muscle development [21,22]. Compared with suckling goats, 373 genes (142 upregulated, 231 downregulated) were identified in early weaned goats, as shown in **S2 and S3 Tables**. In addition, comparisons between the pre- and post- weaning goats revealed 25 altered novel miRNAs, with 17 miRNA levels decreasing and 8 increasing (**S4 Table**).

**Table 1:** Differentially regulated miRNAs identified in goats under weaning.

miRNA	log2(Fold change)	*P*-value	Regulate	Function
chi-miR-206	-4.72	0.0329	down	Development of muscle tissues
chi-miR-99b-3p	-4.6	0.0282	down	Cell proliferation, migration, and invasion regulation
chi-miR-224-3p	-4.2	0.0488	down	Cell proliferation, migration, and invasion regulation
chi-miR-143-5p	-2.06	0.0437	down	Smooth muscle cell proliferation and differentiation
chi-miR-133a-3p	-1.67	0.0002	down	Skeletal muscle proliferation and differentiation
chi-miR-133b	-1.67	0.0002	down	Skeletal muscle proliferation and differentiation
chi-miR-199a-5p	-1.46	0.0000	down	Cell proliferation regulation
chi-miR-100-5p	-1.13	0.0000	down	Cell proliferation, migration, and invasion regulation
chi-miR-10b-5p	-1.09	0.0000	down	Cell proliferation, migration, and invasion regulation
chi-miR-145-3p	-1.06	0.0097	down	Smooth muscle cell proliferation and differentiation
chi-miR-144-5p	1.02	0.0000	up	Anti-tumor
chi-miR-485	1.06	0.0001	up	Suppress cancer progression, reverse chemosensitivity
chi-miR-22-5p	1.07	0.0000	up	Potential diagnostic biomarkers for some cancers
chi-miR-147-5p	1.15	0.0390	up	Regulates macrophage inflammatory responses
chi-miR-671-3p	1.28	0.0017	up	Inhibits epithelial-to-mesenchymal transition
chi-miR-380-3p	1.39	0.0000	up	Represses p53 to control cellular survival
chi-miR-493-5p	1.6	0.0000	up	Tumor suppressor
chi-miR-433	1.72	0.0000	up	Tumor suppressor
chi-miR-545-5p	1.78	0.0499	up	Regulate cell proliferation

### GO classification and KEGG pathways of DEGs

**Fig 1:**
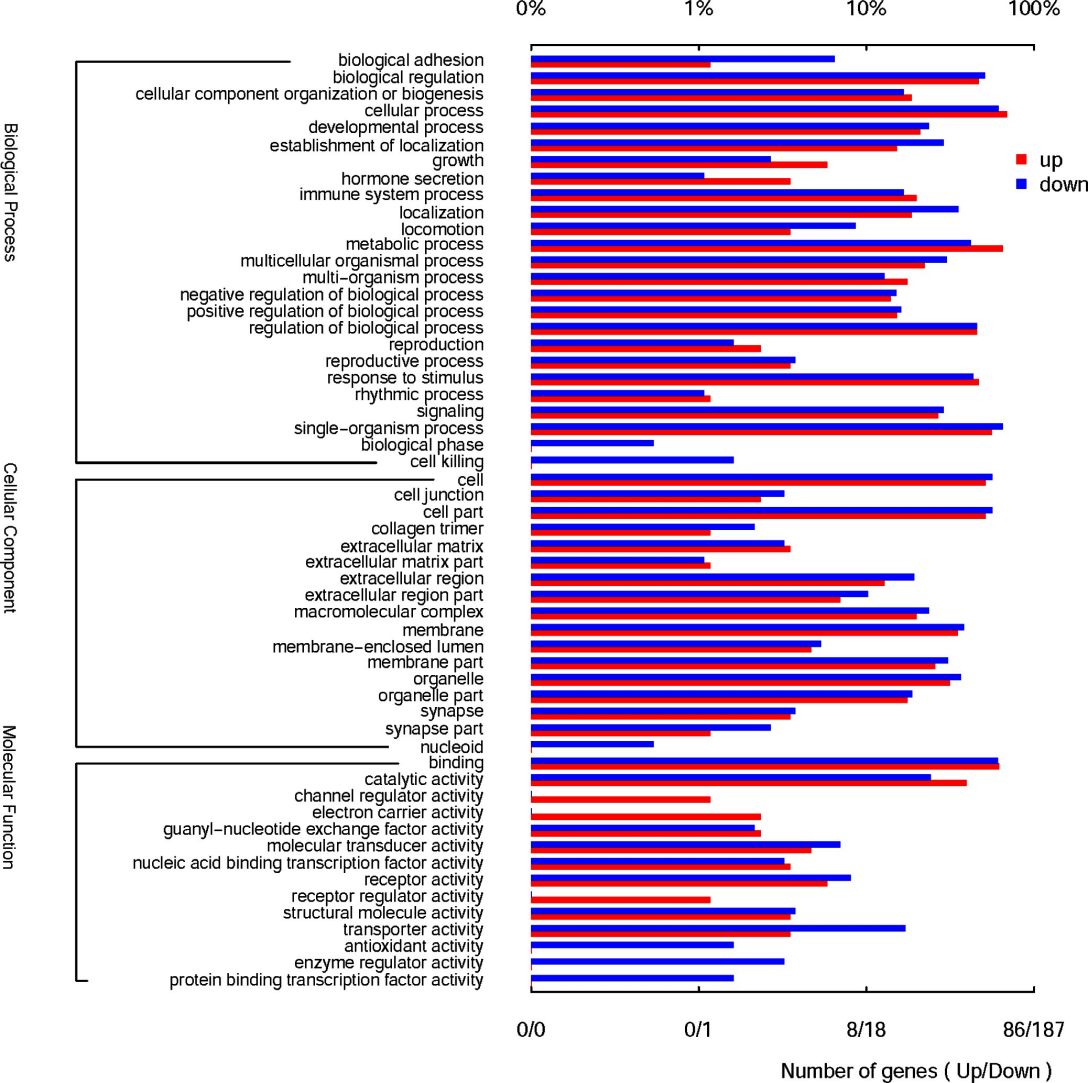
GO enrichment analysis of the differentially expressed mRNAs.

To gain insight into the biological roles of the DEGs, the DEGs were further investigated for GO classification and KEGG enrichment analysis. As shown in **Fig 1**, in terms of biological processes, genes mainly involved in biological adhesion, biological phase, and cell killing were downregulated in goats after weaning. In terms of molecular function, downregulated genes were mainly involved in transporter activity, antioxidant activity, and enzyme regulator activity. This result was consistent with the downregulation of the measured serum antioxidant index (**S5 Table**). The upregulated genes were involved in channel regulator activity and electron carrier activity. In addition, both the up- and downregulated genes function in cellular process, developmental process, and growth in the biological process analysis **(Fig 1)**.

For KEGG analysis, downregulated genes were mainly enriched in salivary secretion, bile secretion, vascular smooth muscle contraction, and calcium signaling pathway among others (**Table 2**). Among them, salivary secretion and bile secretion pathways belong to the digestive system pathways, and the downregulation of this pathway indicated that the digestive system of the lamb was impaired after weaning. Vascular smooth muscle contraction and calcium signaling pathways are involved in bone formation and muscle development. The downregulation of gene expression in these two pathways indicated that the hindered growth of lambs after weaning may be associated with the dysfunction of these two pathways.

**Table 2:** KEGG pathway analysis of the differentially expressed mRNAs.

KEGG pathway	Sample number	*P*-Value	Genes		typeII
			DOWN	UP	
Salivary secretion	8	0.0001	*NOS1*, *CAMP*,*MAP34-B*, *LOC102171106*, *LOC102169231*, *MAP28*, *BAC7*.*5*, *GUCY1A3*		Digestive system
Bile secretion	7	0.0005	*ABCG2*, *LOC102187779*, *LOC102173859*, *ABCC4*, *AQP1*, *SLC22A1*	*BAAT*	Digestive system
Vascular smooth muscle contraction	7	0.0044	*PTGIR*, *CALD1*, *MYLK*, *GUCY1A3*, *ACTA2*,*ACTG2*, *CACNA1D*		Circulatory system
cAMP signaling pathway	9	0.0065	*ABCC4*, *LOC102173859*, *NPY1R*, *FOS*, *PLCE1*, *CACNA1D*,*CNGB1*,*LOC102173859*	*CAMK2B*	Signal transduction
ABC transporters	4	0.0145	*LOC102173859*, *ABCG2*, *LOC102177789*, *LOC102187779*		Membrane transport
RIG-I-like receptor signaling pathway	5	0.0150	*DHX58*	*IRF7*, *ISG15*, *CXCL10*, *DDX58*	Immune system
Cytosolic DNA-sensing pathway	4	0.0183		*IRF7*, *DDX58*,*TIGT2*, *ZBP1*	Immune system
Circadian entrainment	5	0.0227	*NOS1*, *CACNA1D*, *GUCY1A3*, *FOS*	*CAMK2B*	Environmental adaptation
ECM-receptor interaction	4	0.0357	*COL4A1*, *COL1A1*, *ITGA9*	*AGRN*	Signaling molecules and interaction
Arginine biosynthesis	2	0.0386	*NOS1*, *ASS1*		Amino acid metabolism
Calcium signaling pathway	7	0.0483	*NOS1*, *MYLK*, *GNAL*, *HTR2A*, *CACNA1D*	*PLCE1*, *CAMK2B*	Signal transduction

### Integrative analysis of miRNA and mRNA expression data

The potential targets of the 18 miRNAs were predicted using miRanda. A total of 107 genes, belonging to the DEGs and predicted as miRNA targets were identified and listed in **Table 3**. Several genes such as ankyrin repeat and SOCS box protein 14 (*ASB14*) were downregulated in weaned goats, and this gene is a putative target of miR-380-3p. The miRNA gga-miR-206 was repressed in weaned goats (**Table 3**).

**Table 3:** Overlapped genes between putative target genes of miRNAs and differential mRNA genes.

miRNA	Fold change	Mapped targets with mRNA profile
chi-miR-206	0.04	*LOC102168687*, *DHX58*, *LOC108636404*, *AGRN*, *LOC106503599*, *TENM4*, *ALPL*, *NET02*, *ESP8*, *ANK3*, *CD5L*, *STMN2*, *CPLX2*, *ATP2B3*, *CNRIP1*, *ALDH1A3*
chi-miR-99b-3p	0.04	*LOC102168687*, *LOC102186893*
chi-miR-224-3p	0.05	*RSAD2*,*PLCE1*, *ANK3*,*GJA5*,*TEK*,*ADAM22*
chi-miR-143-5p	0.24	*SLFN11*,*TMPRSS2*,*MMP2*,*TENM4*,*CHAC2*,*AQP1*,*GNAL*,*RAB26*,*PHF24*,*ALDH1A3*
chi-miR-133a-3p	0.31	*SLIT1*,*ASB14*, *SEMA3G*, *BCL2L1*, *SH3BGRL2*, *RBPMS2*, *EPB42*, *PHOX2A*
chi-miR-133b	0.31	*SLIT1*, *ASB14*, *SEMA3G*, *BCL2L1*, *SH3BGRL2*, *RBPMS2*, *EPB42*, *PHOX3A*
chi-miR-199a-5p	0.36	*NETO2*, *FMO2*, *ANK3*, *STUM*, *HES4*, *IFI44L*, *LOC106503599*, *CAMKV*
chi-miR-100-5p	0.46	*ST5*
chi-miR-10b-5p	0.47	*FAM11B*, *SH3BGRL2*, *ALPL*, *ANK3*, *CNN1*, *HS6ST2*, *RAB26*
chi-miR-145-3p	0.48	*RNF213*, *LOC106503599*, *TMEM8C*, *HBBBC*, *LOC102175876*, *LOC102176710*, *SOX6*, *THBD*, *ANK3*, *GRPC5C*, *VLDLR*, *SLC17A7*, *FMN1*, *LOC108638214*, *PTPRO*
chi-miR-144-5p	2.03	*SH3BGRL2*, *MAP34-B*, *SYNPO2*
chi-miR-485	2.08	*SLC4A1*, *SOX6*, *ANK3*, *STMN2*, *SH3BGR*, *SLC7A8*, *RNF213*, *LOC102190983*, *LOC106503599*
chi-miR-22-5p	2.10	*CALD1*, *SOX6*, *ANK3*, *VLDLR*, *SH3BGR*, *FMN1*, *STUM*, *ATP2N3*, *XAF1*, *TMEM17*, *CASKIN1*, *TENM4*, *PLCE1*
chi-miR-147-5p	2.22	*SLFN11*, *LOC102172606*, *CSMD1*, *SEMA3G*, *TMEM8C*, *FLT1*, *CALD1*, *DUSP26*, *PHOSPHO1*, *NACC2*, *LOC108633803*, *VLDLR*, *TMOD1*, *HS6ST2*, *TMTC1*,*CNRIP1*, *RAB26*, *NCAM1*, *PHF24*
chi-miR-671-3p	2.43	*SLIT3*, *NTM*, *SEMA3G*, *PDE11A*, *NACC2*, *FAM65C*, *TMOD1*, *HS6ST2*, *HES2*
chi-miR-380-3p	2.62	*ZNFX1*, *LOC102168687*, *SLFN11*, *LOC102180290*, *LOC102186893*, *TMEN17*, *SH3BGRL2*, *SOX6*, *ANK3*, *LOC102190981*, *ARHGEF37*, *ZNF423*
chi-miR-493-5p	3.03	*DHX58*, *ASB14*, *CSMD1*, *LOC108637511*, *LOC102187924*, *ARHGAP5*, *SOX6*, *ANK3*, *RHCE*, *ALB*, *PHF24*
chi-miR-433	3.29	*PLCE1*, *ZNFX1*, *CD5L*, *FAM65C*, *FMN1*
chi-miR-545-5p	3.43	*RNLS*, *PLCE1*, *MGP*, *OLFM4*, *MMRN1*, *HS6ST2*, *TMTC1*, *PDE6A*, *HTR2A*, *ALDH1A3*, *FOS*

**Fig 2:**
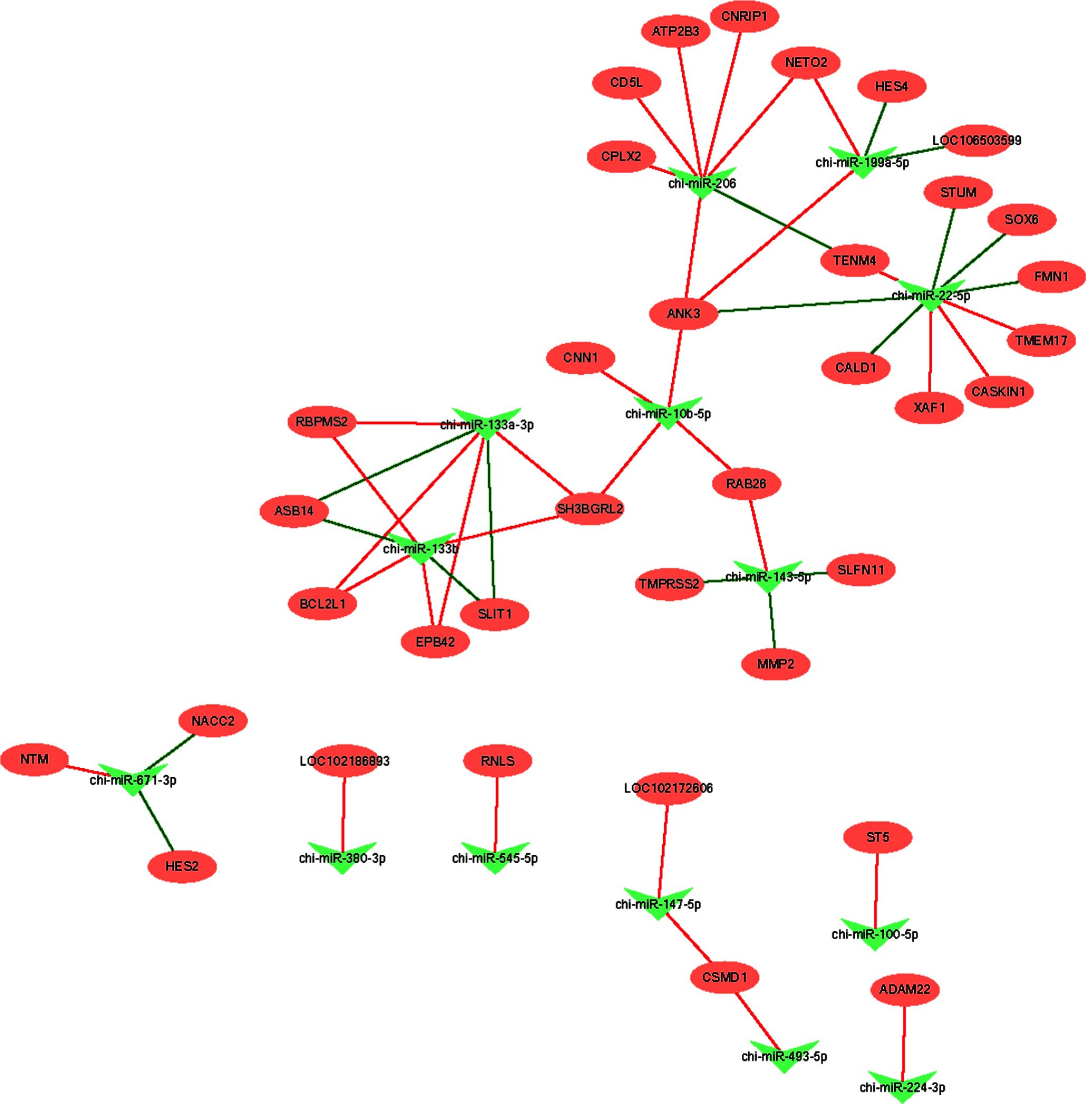
Integrating miRNA and mRNA data to identify anti-correlated target genes associated with weaning stress.

The regulatory network of miRNA-target genes was constructed with the miRNA-target gene pairs by Cytoscape software and the results are shown in **Fig 2**. In this network, chi-miR-206, chi-miR-22-5p, and chi-miR-10b-5p, which regulate 7, 9, and 4 targets, respectively, showed the highest connectivity, whereas *ASB14* and *Schlafen 11* (*SLFN11*) with the highest connectivity were negatively regulated by chi-miR-133a-3p and chi-miR-133b.

### Confirmation of miRNA and mRNA sequencing data

**Fig 3:**
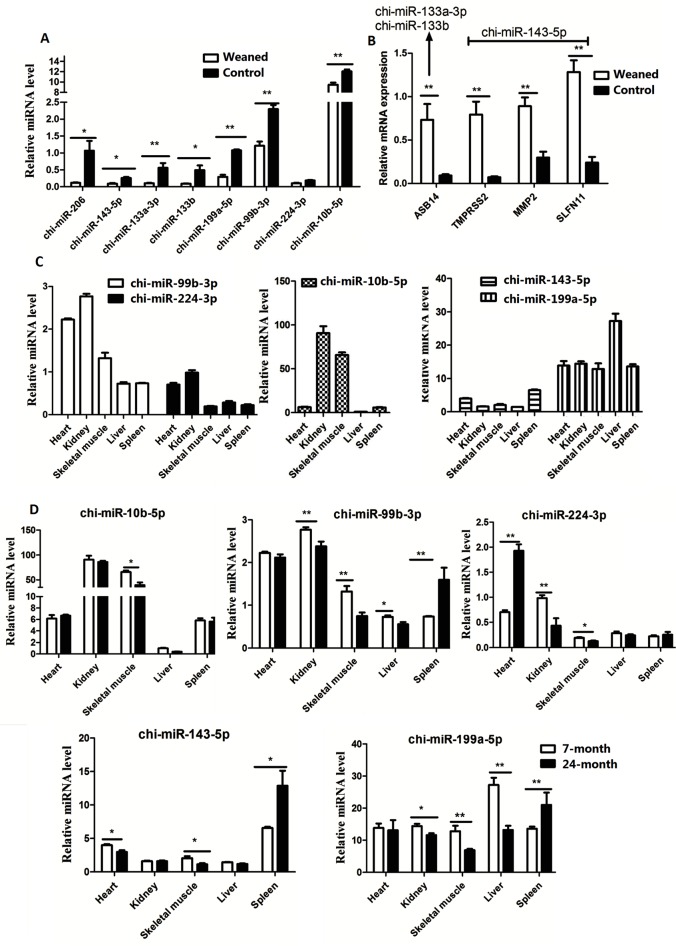
**Analysis the selected 8 differential expressed miRNAs (A) and their targets (B) in the circulating of post-weaned and normal control goats by qRT-qPCR methods. (C) Expression analysis of selected miRNAs in different tissues of 7-month old male goats (D) Expression analysis of selected miRNAs in different tissues of goats between 7-month and 24-month old male goats.** * means *P* < 0.05 and ** means *P* < 0.01.

Eight upregulated miRNAs (chi-miR-206, chi-miR-143-5p, chi-miR-133a-3p, chi-miR-133b, chi-miR-199a-5p, chi-miR-99b-3p, chi-miR-224-3p, and chi-miR-10b-5p) and four altered genes (*ASB14*, *transmembrane serine protease 2 (TMPRSS2)*, *matrix metalloprotease 2 (MMP2)*, and *SLFN11*) in weaned lambs were chosen to validate the sequencing results using RT-qPCR. As shown in **Fig 3A and 3B**, the miRNA and mRNA expressions were similar to those observed using Illumina sequencing analysis. Of the validated DEGs and miRNAs, the mRNA levels of *ASB14* (putative target of chi-miR-133a-3p and chi-miR-133b), *TMPRSS2*, *MMP2*, and *SLFN1*1 (putative targets of chi-miR-143-5p) were significantly increased (*P* < 0.05), whereas the eight selected miRNAs were significantly decreased (*P* < 0.05) in weaned lambs.

### Expression analysis of selected miRNAs in different tissues of goats

In addition to muscle development related miRNAs (chi-miR-206 and chi-miR-133a/b), chi-miR-143-5p, chi-miR-199a-5p, chi-miR-99b-3p, chi-miR-224-3p, and chi-miR-10b-5p, which are involved in cell proliferation [23–27], were downregulated in weaned lambs. The expression profile of these five miRNAs was detected in 7-month old (when the goats are growing rapidly) and 24-month old (when the goats are growing slowly) male Chongming white goats. As shown in **Fig 3C**, the expression of chi-miR-99b-3p and chi-miR-224-3p in heart and kidney tissues was significantly higher (*P* < 0.05) in 7-month old goats compared with those in liver and spleen tissues, whereas chi-miR-99b-3p and chi-miR-224-3p were expressed at high levels (*P* < 0.05) in the skeletal muscles of 7-month old goats. However, chi-miR-199a-5p was specifically expressed in the liver tissues of 7-month old goats. The expression of five miRNAs (chi-miR-99b-3p, chi-miR-224-3p, chi-miR-10b-5p, chi-miR-143-5p, and chi-199a-5p) was significantly decreased (*P* < 0.05) in the skeletal muscles of 24-month old goats compared with that in 7-month old goats, whereas the expression of chi-miR-99b-3p, chi-miR-143-5p, and chi-199a-5p in spleen tissues and the expression of chi-miR-224-3p in heart tissues was significantly increased (*P* < 0.01) in 24-month old goats (**Fig 3D**). In addition, the expression of chi-miR-99b-3p, chi-miR-224-3p, and chi-199a-5p in kidney tissues and the expression of chi-miR-99b-3p and chi-199a-5p were significantly decreased (*P* <0.05) in liver tissues of 24-month old goats (**Fig 3D**).

## Discussion

The present study analyzed the mRNA and miRNA transcriptome of early weaning goats together with miRNA target predictions to elucidate the expression dynamics of mRNA and miRNA and the interplay between genes and miRNAs under weaning stress. A total of 18 miRNAs and 373 genes were significantly altered in the blood of goats in response to weaning stress, and most of the altered miRNAs were downregulated. Among DEGs, 107 overlapped genes were potential targets of the altered miRNAs. In the next step, a global interaction network was constructed by combining differentially expressed miRNAs and mRNAs. Some of the downregulated miRNAs (miR-206, miR-133a/b, and miR-10b-5p) were included in the altered miRNA lists reported by Tao et al. in weaned piglets [12]; however, the expression results reported in that study are the opposite from our results. Although the reasons are unknown, the discrepancy may due to species specific responses and species sensitivity to stress.

In the present study, miRNAs related to cell proliferation (chi-miR-99b-3p, chi-miR-224, chi-miR-143-5p, and chi-miR-10b-5p) and muscle development (chi-miR-206 and chi-miR-133a/b) were significantly altered in post-weaning goats. This result may provide important information regarding weaning stress-induced damage in animals. The miR-206 and miR-133a/b, which are members of the miR-1 family, are specifically expressed in vertebrate skeletal muscle and play important regulatory roles in skeletal muscle cell proliferation and differentiation, suggesting their potential as biomarkers and therapeutic targets in skeletal muscle diseases [28,29]. Local injection of miR-1, miR-133, and miR-206 promotes muscle regeneration in a rat skeletal muscle injury model [30]. Furthermore, a mutation in the 3'-UTR of the *myostatin* gene in Texel sheep is responsible for the muscular hypertrophy phenotype of Texel sheep by creating a target site for miR-206 and miR-1, indicating the regulatory of miR-206 in muscle development in sheep [21]. In the present study, we confirmed that chi-miR-206 and chi-miR-133a/b were highly expressed in the skeletal muscles of 7-month old goats (when the goats are growing rapidly, **S3 Fig**). The downregulation of chi-miR-206 and chi-miR-133a/b may indicate depressed muscle development function in weaned goats.

Other downregulated miRNAs (chi-miR-99b-3p, chi-miR-224, chi-miR-143-5p, chi-miR-10b-5p, and chi-miR-199a-5p) are involved in cell proliferation, apoptosis, and differentiation. For example, low levels of miR-99b-5p and miR-203a-3p are observed in gastric cancer, and upregulation of miR-99b-5p/203a-3p inhibits cell proliferation and cell cycle progression in gastric cancer [31]. Overexpression of miR-99b-3p inhibits cell proliferation in oral squamous cell carcinoma [32]. However, conflicting results are reported in normal mouse mammary gland cells, as miR-99a and miR-99b overexpression promotes cell proliferation, migration, and fibronectin expression by negatively regulating the expression of E-cadherin [23]. Taken together, the results described above indicated that miR-99b has distinct functions in different cell types.

The miR-224 upregulation promotes cell proliferation and invasion in some types of cancer cells, including non-small cell lung cancer [33] and colorectal cancer [34]. In smooth muscle cells, miR-10b is upregulated in vascular smooth muscle cells isolated from atherosclerotic patients and promotes cell proliferation by regulating the Akt pathway [25]. The miR-143 and miR-145 are involved in the modulation of smooth muscle cell development, and the miR-143/145 cluster promotes cell proliferation and migration in pulmonary arterial smooth muscle cells [35], whereas it represses the same functions in vascular smooth muscle cells to reverse cell proliferation in intracranial aneurysms [36]. The miR-143 regulates skeletal muscle differentiation, and overexpression of miR-143-3p increases, whereas inhibition of miR-143-3p decreases muscle fiber differentiation in porcine skeletal muscle satellite cells (MSCs) [37]. High levels of miR-143 are associated with the bovine MSCs differentiation process [38]. The miR-143 upregulation inhibits MSC proliferation and differentiation, whereas miR-143 downregulation inhibits cell proliferation and promotes differentiation by regulating insulin like growth factor binding protein 5 [38]. In the current study, chi-miR-99b-3p, chi-miR-224, chi-miR-143-5p, and chi-miR-10b-5p were significantly downregulated in goats in response to weaning stress, suggesting that cell proliferation activity was suppressed in post-weaning goats. Furthermore, assessment of the expression profiles of these downregulated miRNAs in 7-month old Chongming white goats showed that chi-miR-99b-3p, chi-miR-224, chi-miR-143-5p, and chi-miR-10b-5p were highly expressed in muscle tissues (heart, kidney, or skeletal muscle), suggesting that these miRNAs play important roles in goat muscle development. The findings that muscle development-related miRNAs (chi-miR-206 and chi-miR-133a/b) and the potential muscle development-associated miRNAs (chi-miR-99b-3p, chi-miR-224, chi-miR-143-5p, and chi-miR-10b-5p) were all significantly downregulated in early weaning goats provided important information for weaning induced growth inhibition in domestic animals. Notably, the functions of most changed miRNAs are rarely studied in animals. The above speculation is mainly based on the reports of human, and further researches are needed to study the specific functions of these miRNAs in goats.

KEGG pathway analysis of the DEGs was performed to evaluate the functional changes in goats in response to weaning stress. The results showed that the downregulated genes were mainly enriched in salivary secretion and bile secretion, which belong to the digestive system pathway. Repression of this pathway in weaning goats indicated that the digestive system of the lamb was impaired after weaning. In the current study, vascular smooth muscle contraction and the calcium signaling pathway, which are involved in bone formation and muscle development, were suppressed by reduced gene expression in response to weaning stress. Furthermore, the downregulation of gene expression associated with vascular smooth muscle contraction was consistent with the downregulation of chi-miR-143/145, suggesting the potential role of chi-miR-143/145 in the regulation of vascular smooth muscle development in weaning goats. A total of 107 overlapped genes were identified by comparative analysis between the putative targets of the changed miRNAs and the DEGs. Some of these were negative correlated with the altered miRNAs, such as *ASB14* (putative target of chi-miR-133a-3p and chi-miR-133b), *TMPRSS2*, *MMP2*, and *SLFN11* (putative targets of chi-miR-143-5p), and this was confirmed by RT-qPCR. The results suggested that these genes were potential targets of the miRNAs, although further experiments are needed to confirm these findings. In a recent study, altered expression of ASB14 was observed in ischemic cardiomyopathy heart failure. Biological process functional analysis indicated that ASB14 may be associated with heart failure through the regulation of protein ubiquitination, which requires experimental validation [39]. TMPRSS2 functions in prostate carcinogenesis [40] and contributes to the spread of several viruses [41]. High levels of MMP-2 are associated with tumor progression, including cancer cell proliferation, invasion, metastasis, and progression [42,43]. In addition, the SLFN family is involved in development, immune responses, and cell proliferation [44]. High levels of SLFN11 sensitize cancer cells to DNA damaging agents by suppressing checkpoint maintenance and homologous recombination repair [45]. Taken together with previous findings, the present results showing miRNA alterations and the upregulation of the potential targets suggested their involvement in cell proliferation and differentiation, as the DEGs were mostly enriched in cellular processes, developmental processes, and growth.

In the present study, to gain insight into the functional changes in goats in response to weaning stress, we focused on miRNAs and mRNA profiles in the whole blood of post-weaning goats by deep sequencing. We found that the genes regulated by altered miRNAs were mostly involved in cell proliferation and differentiation, which provided useful knowledge on miRNA regulatory mechanisms in post-weaning goats. The skeletal muscle development-related miRNAs (chi-miR-206 and chi-miR-133a/b) were significantly downregulated in post-weaning goats, and these findings provided important information regarding weaning stress-induced growth retardation in livestock. Most of the cell proliferation-associated miRNAs, such as chi-miR-99b-3p, chi-miR-224, chi-miR-143-5p, and chi-miR-10b-5p, were also downregulated in goats in response to weaning stress, suggesting that post-weaning repressed cell proliferation in goats. These downregulated miRNAs were highly expressed in the muscle tissues of goats during the rapid growth period, and this provided new insight into the mechanism of muscle development of goats, although further details remain to be elucidated in the future. The significantly changed miRNAs can be used as potential biomarkers to assess the severity of weaning stress in the goat production. Then the goat production mode can be improved correspondingly by reducing the weaning stress via feed additive supplementation or management improvement.

## Supporting information

S1 Fig Workflow of the study design and analyses. (TIFF)

S2 Fig Hierarchical clustering of differentially expressed miRNAs induced by weaning stress. W: weaned, C: control.
(TIF)

S3 Fig Analysis of muscle development associated miRNAs. (A) Expression analysis of muscle development associated miRNAs in different tissues of 7-month old male goats; (B) Expression analysis of muscle development associated in different tissues of goats between 7-month and 24-month old male goats. * means *P* < 0.05 and ** means *P* < 0.01.
(TIFF)

S1 Table Primers used for qRT-PCR. (DOCX)

S2 Table Summary of differentially regulated miRNAs and mRNAs. (DOCX)

S3 Table Differential expression genes between weaned and control goats. (XLSX)

S4 Table Altered novel miRNAs between weaned and control goats. (DOCX)

S5 Table Analysis of serum biological index between weaned and control goats^#^. #Serum samples were isolated, and antioxidant index, including superoxide dismutase (SOD) and reduced glutathione (GSH) were analyzed according to the protocol provided by the manufacturer (Nanjing Jiancheng Bioengineering Institute, Nanjing, China). Triglyceride (TG), albumin (ALB), and cholesterol (T-CHO) concentrations were determined on the automatic biochemical analyzer (AU5800, Beckman, MN, USA). ^a,c^ Mean values with unlike letters were significantly different (*P* < 0.01).
(DOCX)
