# Supplementary figures and images for: Altered expression of miRNAs and mRNAs reveals the potential regulatory role of miRNAs in the developmental process of early weaned goats
Source: PLoS One. 2019 Aug 8;14(8):e0220907. doi: 10.1371/journal.pone.0220907 (PMC6687162; doi:10.1371/journal.pone.0220907)

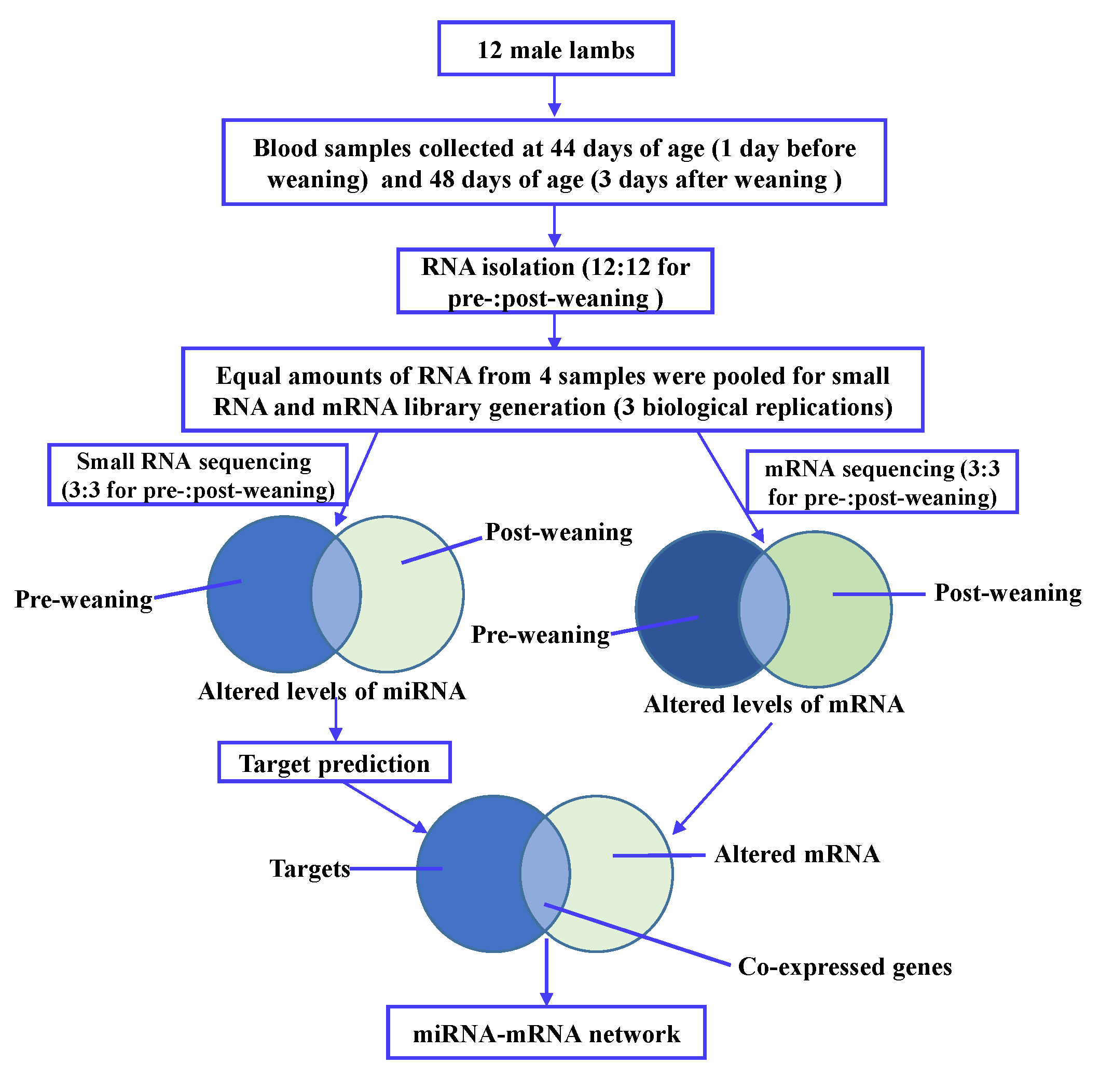

Supplement: S1 Fig — (TIFF) [file pone.0220907.s001.tiff]

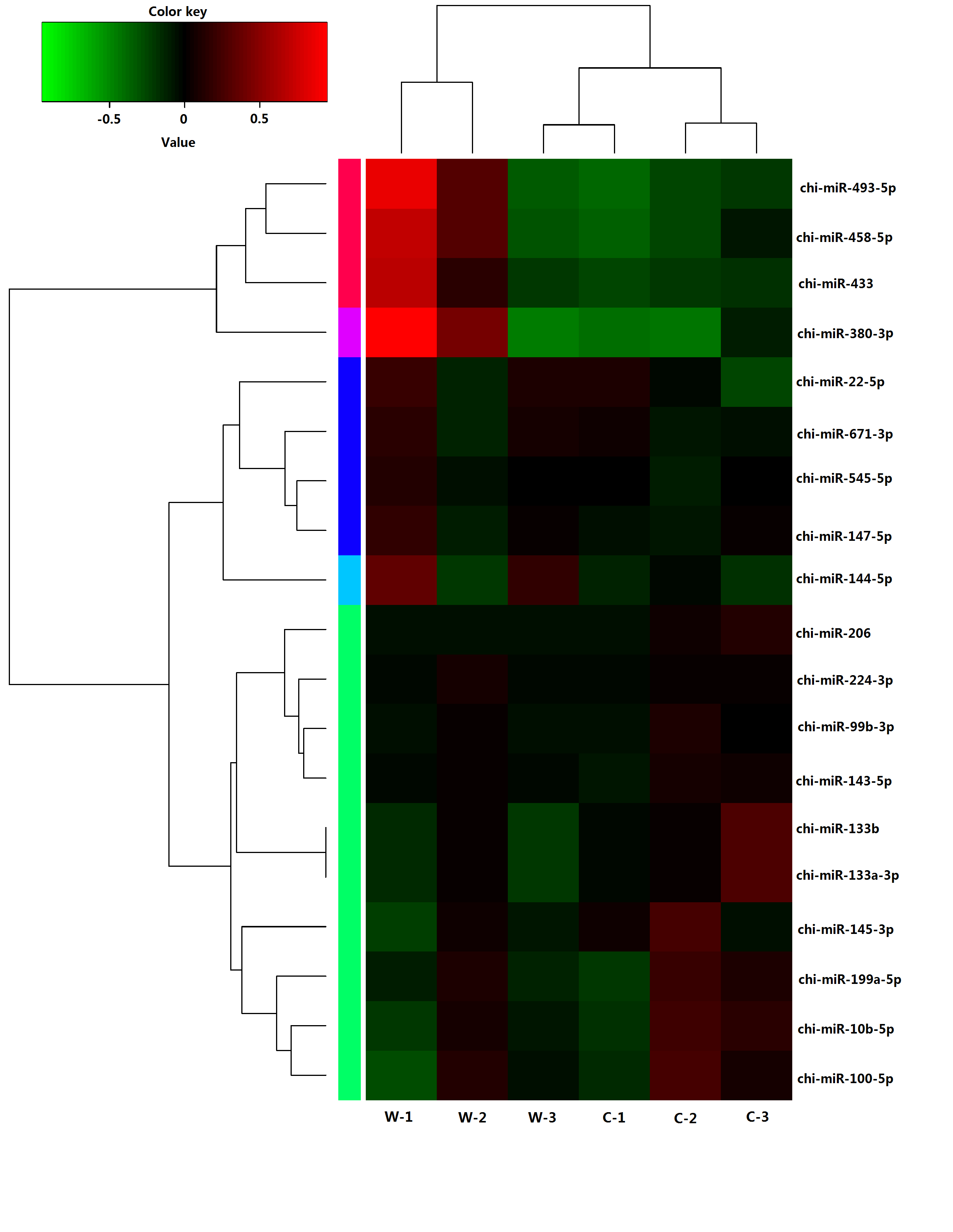

Supplement: S2 Fig — W: weaned, C: control. (TIF) [file pone.0220907.s002.tif]

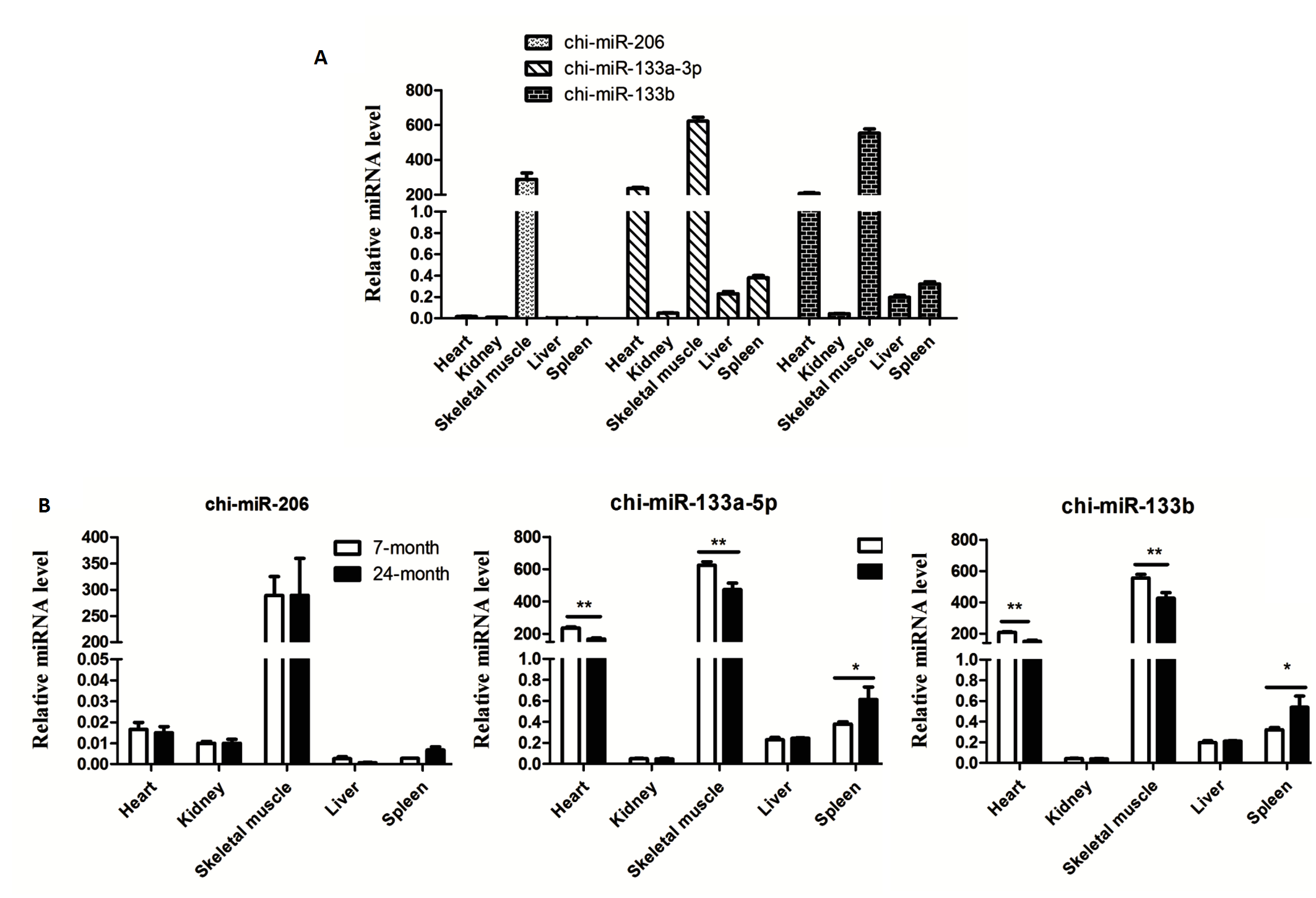

Supplement: S3 Fig — (A) Expression analysis of muscle development associated miRNAs in different tissues of 7-month old male goats; (B) Expression analysis of muscle development associated in different tissues of goats between 7-month and 24-month old male goats. * means P < 0.05 and ** means P < 0.01. (TIFF) [file pone.0220907.s003.tiff]
