# Supplementary material for: Altered expression of miRNAs and mRNAs reveals the potential regulatory role of miRNAs in the developmental process of early weaned goats
Source: PLoS One. 2019 Aug 8;14(8):e0220907. doi: 10.1371/journal.pone.0220907 (PMC6687162; doi:10.1371/journal.pone.0220907)
Supplement: S1 Table — (DOCX) [file pone.0220907.s004.docx]

**S1 Table. Primers used for qRT-PCR**

| **Gene Symbol** | **GeneBank** | **Forward primer** | **Reverse primer** | **Product length(bp)** |
| --- | --- | --- | --- | --- |
| **mRNA** |  |  |  |  |
| SLFN11 | XM_005693205.3 | AGTATATCCCTGCATTTGCG | TTTCAAAGAGTCACGGTCAAC | 116 |
| TMPRSS2 | XM_005675629.3 | GAGTGCTGGCTGGAGATAA | TATGTTCCCAGGGCTCTGTCTA | 118 |
| ASB14 | XM_013973561.2 | CTCGATCAGAGAATCCGCA | GCACTCAGAAGCAGCCTA | 104 |
| MMP2 | XM_005691985.3 | GAAGGACTCAGGTTGTCTGAA | TGACAGAGGAGGGACATAACTA | 99 |
| GAPDH | XM_005680968.1 | TGACCTTCACTACATGGTCT | ACTTGATGTTGGCAGGAT | 146 |
| **miRNA** |  |  |  |  |
| U6 |  | CAAGGATGACACGCAAATTCG |  |  |
| chi-miR-206 |  | TGGAATGTAAGGAAGTGTGTG |  |  |
| chi-miR-143-5p |  | GGTGCAGTGCTGCATCTCT |  |  |
| chi-miR-133a-3p |  | TTTGGTCCCCTTCAACCAGC |  |  |
| chi-miR-133b |  | TTTGGTCCCCTTCAACCAGC |  |  |
| chi-miR-199a-5p |  | CCCAGTGTTCAGACTACCTG |  |  |
| chi-miR-99b-3p |  | CAAGCTCGTGTCTGTGGGT |  |  |
| chi-miR-224-3p |  | AAATGGTACCCTAGTGACTACA |  |  |
| chi-miR-10b-5p |  | TACCCTGTAGAACCGAATTTGT |  |  |
