# Supplementary material for: Altered expression of miRNAs and mRNAs reveals the potential regulatory role of miRNAs in the developmental process of early weaned goats
Source: PLoS One. 2019 Aug 8;14(8):e0220907. doi: 10.1371/journal.pone.0220907 (PMC6687162; doi:10.1371/journal.pone.0220907)
Supplement: S2 Table — (DOCX) [file pone.0220907.s005.docx]

**S2 Table. Summary of differentially regulated miRNAs and mRNAs**

| Item | Known miRNA | Novel miRNA | mRNA |
| --- | --- | --- | --- |
| Increased | 8 | 8 | 142 |
| Decreased | 10 | 17 | 231 |
| No change | 389 | 720 | 9611 |
| Total | 407 | 745 | 9984 |
