# Supplementary material for: Altered expression of miRNAs and mRNAs reveals the potential regulatory role of miRNAs in the developmental process of early weaned goats
Source: PLoS One. 2019 Aug 8;14(8):e0220907. doi: 10.1371/journal.pone.0220907 (PMC6687162; doi:10.1371/journal.pone.0220907)
Supplement: S4 Table — (DOCX) [file pone.0220907.s007.docx]

**S4 Table. Altered novel miRNAs between weaned and control goats**

| **miR_name** | **Fold change**  **(Weaned/Control)** | ***P* value** | **Novel miRNA sequence** |
| --- | --- | --- | --- |
| NC_030830_1_2176 | 0.0300 | 0.0225 | uugguccccuucaaccagc |
| NC_030826_1_1735 | 0.2031 | 0.0000 | gaggguuuggguuuggucguggga |
| NC_030809_1_185 | 0.2736 | 0.0088 | aaaaggucauucggguuuucc |
| NC_030815_1_844 | 0.3015 | 0.0364 | ugaauucugguucugaucacu |
| NC_030820_1_1263 | 0.3035 | 0.0001 | uugguccccuucaaccagcugu |
| NC_030820_1_1264 | 0.3035 | 0.0001 | uugguccccuucaaccagcugu |
| NC_030831_1_2199 | 0.3035 | 0.0001 | uugguccccuucaaccagcugu |
| NC_030809_1_151 | 0.3164 | 0.0132 | auagaggggccccccaggcu |
| NC_030812_1_479 | 0.3209 | 0.0126 | aagaacuuaaaugaacuuuuug |
| NC_030817_1_1012 | 0.3789 | 0.0001 | aaaaauugaacaaacuuguugg |
| NC_030822_1_1364 | 0.4323 | 0.0000 | aacccguagauccgaacuugu |
| NC_030824_1_1546 | 0.4353 | 0.0153 | aaaaggucauucggguuuucc |
| NC_030809_1_144 | 0.4444 | 0.0000 | uacccuguagaaccgaauuugu |
| NC_030820_1_1251 | 0.4730 | 0.0042 | ugaggauuuugcuuguuucau |
| NC_030826_1_1709 | 0.4763 | 0.0387 | acuuugaccuguaacccaucugg |
| NC_030808_1_82 | 0.4796 | 0.0030 | gcgacccauacuugguuucaga |
| NC_030836_1_2445 | 0.4965 | 0.0010 | cagggagggcugggggcuuggg |
| NC_030811_1_348 | 2.0139 | 0.0109 | aaaucuacagauugucgaaaugg |
| NC_030825_1_1611 | 2.0420 | 0.0130 | uuugcugcgacgccugcuggga |
| NC_030828_1_1879 | 2.1287 | 0.0000 | uaugugggacgguaaaccgcu |
| NC_030815_1_796 | 2.1585 | 0.0138 | ucccgguccuucacuagcacug |
| NC_030828_1_1881 | 2.3784 | 0.0000 | uauguaaugugguccacgucu |
| NC_030828_1_1916 | 2.4967 | 0.0071 | ugaccuuccugcaucccguuc |
| NC_030809_1_101 | 3.3636 | 0.0014 | uaauauaggacuuccccggugg |
| NC_030818_1_1104 | 5.8159 | 0.0007 | ucggggucggaggaagguucu |
