# Supplementary material for: Altered expression of miRNAs and mRNAs reveals the potential regulatory role of miRNAs in the developmental process of early weaned goats
Source: PLoS One. 2019 Aug 8;14(8):e0220907. doi: 10.1371/journal.pone.0220907 (PMC6687162; doi:10.1371/journal.pone.0220907)
Supplement: S5 Table — #Serum samples were isolated, and antioxidant index, including superoxide dismutase (SOD) and reduced glutathione (GSH) were analyzed according to the protocol provided by the manufacturer (Nanjing Jiancheng Bioengineering Institute, Nanjing, China). Triglyceride (TG), albumin (ALB), and cholesterol (T-CHO) concentrations were determined on the automatic biochemical analyzer (AU5800, Beckman, MN, USA). a,c Mean values with unlike letters were significantly different (P < 0.01). (DOCX) [file pone.0220907.s008.docx]

**S5 Table Analysis of serum biological index between weaned and control goats#**

| **Item** | **Control** | **Weaned** |
| --- | --- | --- |
| **GSH (μmol/L)** | 55.54±9.72^a^ | 36.44±10.43^a^ |
| **SOD (U/mL)** | 18.54±1.04^a^ | 11.37±2.15^c^ |
| **ALB (g/L)** | 21.86±2.63 | 21.76±4.57 |
| **T-CHO (nmol/L)** | 8.76±2.64^a^ | 4.76±2.67^c^ |
| **TG (mmol/L)** | 1.03±0.22^a^ | 0.98±0.22^a^ |

#Serum samples were isolated, and antioxidant index, including superoxide dismutase (SOD) and reduced glutathione (GSH) were analyzed according to the protocol provided by the manufacturer (Nanjing Jiancheng Bioengineering Institute, Nanjing, China). Triglyceride (TG), albumin (ALB), and cholesterol (T-CHO) concentrations were determined on the automatic biochemical analyzer (AU5800, Beckman, MN, USA).

a,c Mean values with unlike letters were significantly different (P < 0.01).
